# The use of tissue adhesive as an adjunct to wound closure in knee arthroplasty does not reduce wound ooze

**DOI:** 10.1186/s43019-020-00073-0

**Published:** 2020-10-31

**Authors:** Mohamed A. Khalefa, Lindsay K. Smith, Riaz Ahmad

**Affiliations:** 1 Trauma and Orthopaedics, Weston General Hospital, Weston-super-Mare, UK; 2 Trauma and Orthopaedics, Cairo University Hospitals, Cairo, Egypt; 3 Centre for Health and Clinical Research, University of the West of England, Bristol, UK; 4 Orthopaedic Department, Weston General Hospital, Weston-super-Mare, BS23 4TQ UK; 5 Musculoskeletal Research Unit, University of Bristol at Southmead Hospital, Bristol, UK

**Keywords:** Wound ooze, Total knee replacement, Arthroplasty, Butyl cyanoacrylate, Staples, Skin tissue adhesive

## Abstract

**Introduction:**

Persistent wound ooze has been associated with prolonged length of hospital stay and increased risk of infection. Recently, the use of tissue adhesive after hip and knee arthroplasty has been described. We believe that knee arthroplasty wounds exhibit different behavior compared to hip arthroplasty due to the increased wound-margin tension associated with knee flexion.

**Patients and methods:**

Forty-three patients undergoing total knee arthroplasty (TKA) by a single surgeon were studied. All wounds were closed using staples with or without tissue adhesive. Post-operatively, the wounds were reviewed daily for ooze. Dressings were changed only if soaked > 50% or if there was persistent wound discharge of more than 2 × 2 cm at 72 h.

**Results:**

There were 21 patients in the tissue adhesive (group 1), 22 in the non-tissue adhesive (group 2) with the average age for group 1 of 72.2 years and for group 2 of 69.3 years. The median length of stay for both groups was 4 days (range of 3–7 days for group 1 and 2–6 days for group 2) (*P* = 0.960). The tissue adhesive group showed a statistically significant reduction in wound ooze on day 1 (*P* = 0.019); however, the difference was not significant on the following days. The median for the number of dressing changes for group 1 was zero changes and for group 2, one change. This was not statistically significant (*P* = 0.112). No complications were observed in both groups and there were no reactions to the tissue adhesive.

**Conclusion:**

The data from this case series suggest that the use of tissue adhesive may reduce wound ooze on day 1 only. The latter is most likely due to significant tensile forces to which the knee arthroplasty wound is subjected in the immediate post-operative rehabilitation. Further, the cost of tissue adhesive is not offset by reduced dressing changes or length of hospital stay.

## Highlights

- There is little evidence on the best closure method in TKA and no evidence on the use of tissue adhesives as adjuvant to standard closure techniques
- The anterior midline incision is subjected to significant tensile forces during knee flexion which can have disruptive effects on wound margins
- The use of b utyl cyanoacrylate adhesive does not reduce wound ooze or hospital stay when used as an adjunct to staples post TKA

## Introduction

Wound ooze following total hip arthroplasty (THA) and total knee arthroplasty (TKA) remains a topic with controversies, debates and ongoing research surrounding the issue. Persistent wound ooze has been associated with prolonged hospital length of stay (LOS) and increased risk of infection with its catastrophic outcomes [1–3]. Orthopedic surgeons have been striving over the past few decades to identify factors associated with persistent wound ooze and comparing techniques in wound closure and dressing to overcome it, but there is little evidence as to which is the most effective method [4].

Enhanced recovery programs in orthopedics have been well-established and led to rapid rehabilitation and shorter length of hospital stay for arthroplasty patients. Tissue-sparing, minimally invasive, surgical techniques are rapidly evolving and draw more attention to the types of dressings and methods of wound closure utilized to maximize speed of closure in theaters, minimize wound-related complications and expedite patient discharge [5, 6].

Tissue adhesive has many successful applications in different surgical specialties, either as a primary method of closure or as a supplement to sutures [7–10], but requires caution when used in areas of the body that are subjected to tension, due to the risk of wound breakdown [11]. Surgical wounds after THA and TKA have been closed using either sutures or staples but there is no evidence in the literature to favor one method over the other [12]. More recently, tissue adhesives have been introduced with success in arthroplasty, with reduction in wound closure time and ooze in THA, but this has not been proven in TKA where it resulted in prolonged wound ooze [6, 13–16].

The anterior midline incision is the commonest skin incision utilized in TKA and is subjected to significant tensile forces during the various degrees of knee flexion required in the immediate post-operative period due to the nature of rehabilitation process [17, 18]. These forces can have a disruptive effect on the wound margins, and tissue adhesive, if used, may not prevent persistent wound ooze. Unlike in TKA, there are not such disruptive forces in play in the skin incisions used in THA.

At our institution a midline skin incision is used for TKA and the skin is closed with staples. However, due to concerns regarding wound ooze, and owing to the success of tissue adhesive in reducing wound ooze in THA patients [12–15], we started using the tissue adhesive butyl cyanoacrylate (BCA), LiquiBand® (Advanced Medical Solutions Ltd., Winsford, UK). We subsequently undertook a review of the outcome in our series, comparing the cohort of patients who had had their wounds closed with staples compared to those who had had their wound closure with staples and tissue adhesive.

## Patients and methods

All patients undergoing cemented TKA under the care of the senior author, between June 2017 and February 2018, were identified. The data was analyzed retrospectively by interrogation of a database that was maintained due to wound-ooze concerns in our arthroplasty patients.

**Fig. 1:**
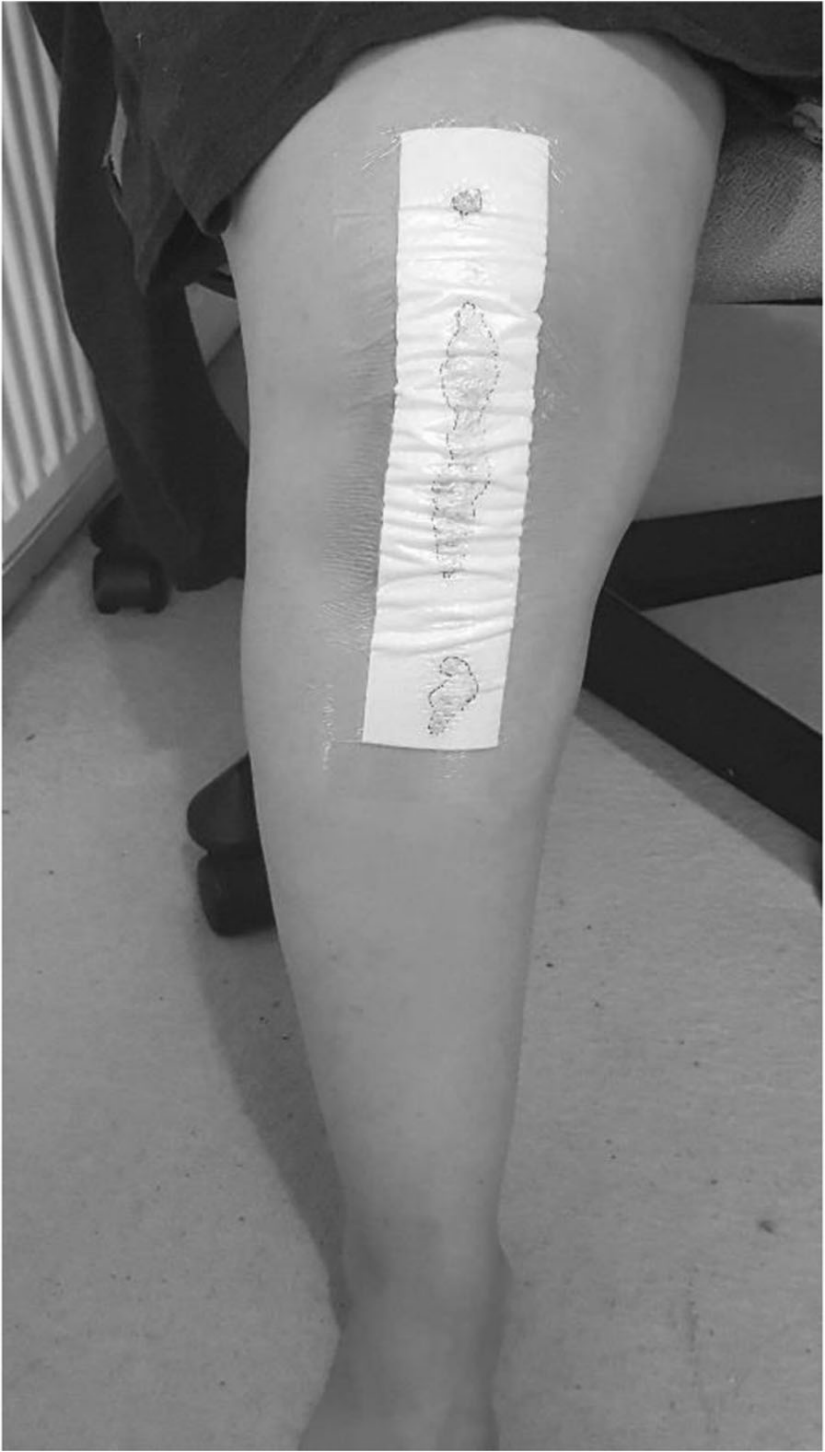
Clinical photograph showing post-operative wound ooze (edges marked)

All patients received the same preoperative and post-operative care, including the administration of peri-operative antibiotics and thromboprophylaxis. All long-term anticoagulation medicines (clopidogrel) were withheld for 7 days prior to surgery and restarted after the wound was dry. One gram of tranexamic acid was given intravenously (IV) at induction of surgery and a further dose of 500 mg was given orally 6 h post-operative. All surgeries were performed using an anterior midline skin incision and medial parapatellar arthrotomy, without the use of a tourniquet. The skin incision was marked 8 cm above and below the upper and lower pole of the patella, respectively, thus the total length of the incision was 21 cm. A Vanguard Cruciate Retaining (CR) implant (Zimmer Biomet Inc., Warsaw, IN, USA) was used for primary knee arthroplasty and the Vanguard 360 Revision implant (Zimmer Biomet Inc., Warsaw, IN, USA) was used for revision arthroplasty. Meticulous hemostasis was carried throughout the operation and before closure. A one-hundred-milliliter mix of ropivacaine 2% plus 1 ml of 1/1000 adrenaline and 40 mg ketorolac was used as local infiltration to the capsule and retinaculum plus extra 50 ml of the same mix without adrenaline to the fat and subcutaneous tissue. All wounds were closed in layers by the senior author (RA). The extensor mechanism was closed using size 2 Vicryl, with equally spaced, three interrupted sutures along the medial border of patella, followed by a continuous suture from the proximal to distal extent of the arthrotomy. The fat was closed with size 0 Vicryl interrupted sutures and the subcutaneous layer with size 0 Vicryl continuous sutures. The skin was closed with staples. LiquiBand® (Advanced Medical Solutions Ltd., Winsford, UK) when used was applied topically on the entire length of the incision in a single layer allowing 20 s for polymerization before the application of a standard 30 × 10-cm Hydrofilm Plus® (Paul Hartmann Ltd., Heywood, UK) adhesive dressing with an absorbent pad (Fig. 1). A compression bandage, in the form of two layers of wool and crepe, was applied and left until the next morning. No drains were used in any of the patients. Thromboprophylaxis was in the form of tinzaparin 4500 units in a single dose on the night of the operation (day 0) followed by apixaban 2.5 mg once daily on the following morning for 10 days. Mechanical thromboprophylaxis was used in form of thrombo-embolic deterrent stockings (TEDs) and foot pumps to all patients unless contraindicated. All patients underwent an enhanced recovery program with immediate full weightbearing and range of motion (ROM) exercises under physiotherapy guidance. All patients had the staples removed at 14 days and were followed up at 6 weeks and 6 months after the index procedure.

**Fig. 2:**
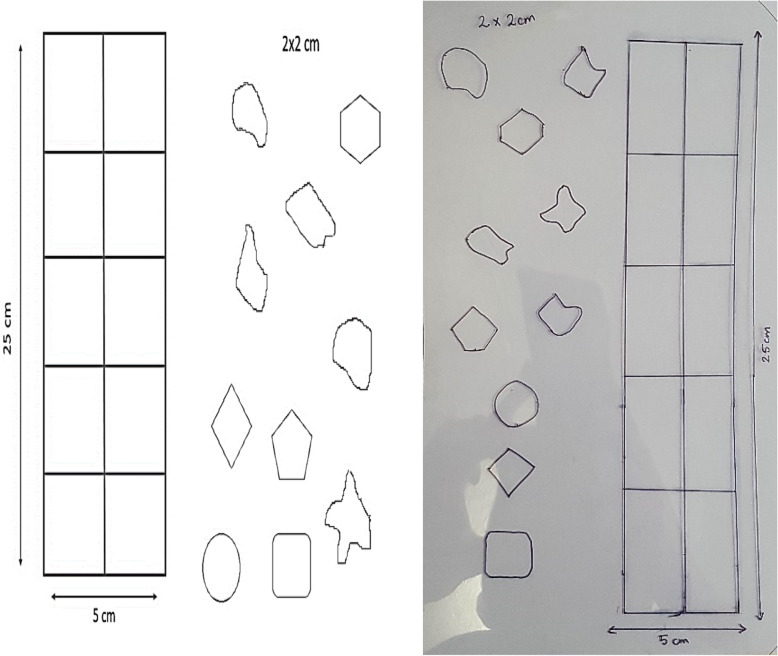
Template used to quantify wound ooze. Left: schematic diagram. Right: actual clear template used

**Fig. 3:**
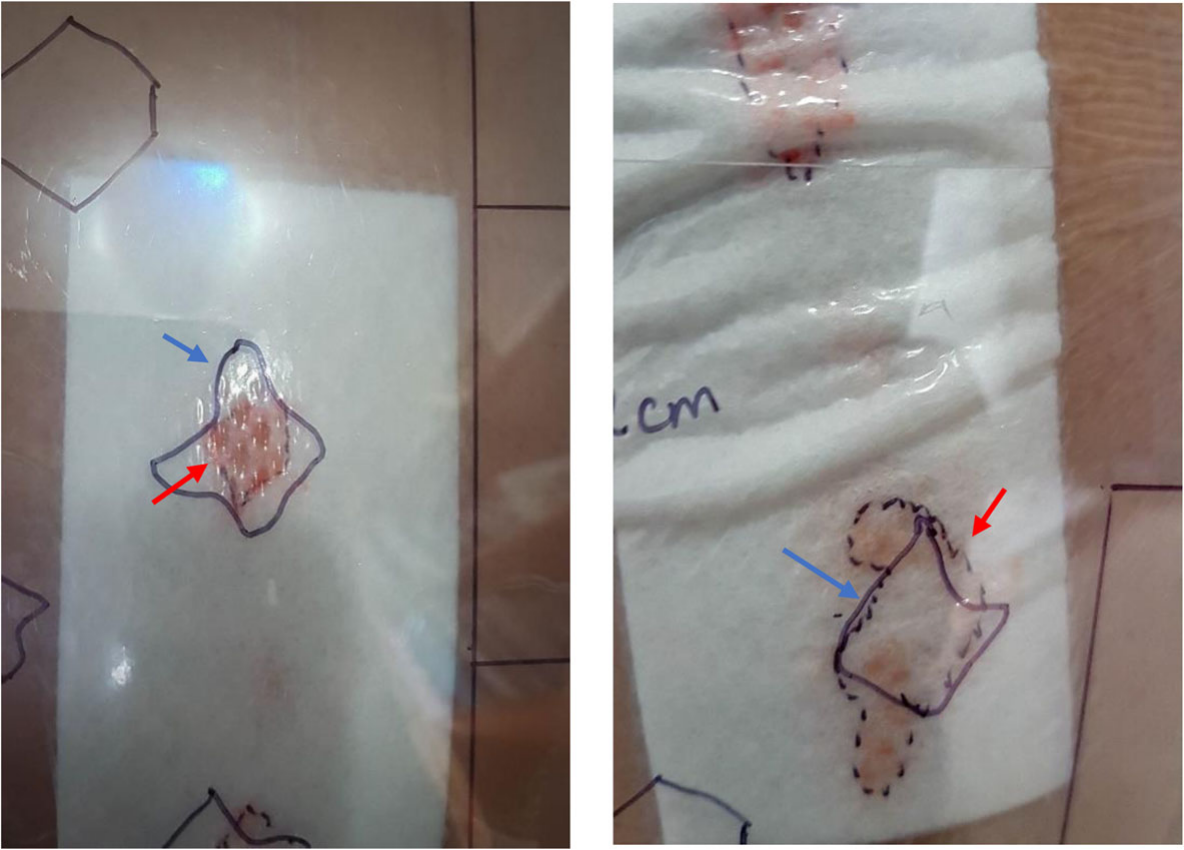
Clear template with a shape of 2 × 2 cm overlying a spot of wound ooze. Left: smaller than 2 x 2 cm. Right: larger than 2 x 2 cm. Red arrow indicates edge of ooze. Blue arrow indicates edge of the shape on the template

The data was collected by an observer (MAK) blinded to the use of wound adhesive, using a specifically designed proforma (Table 1). A quantitative assessment of wound ooze was done on a daily basis for the need of dressing change, according to our hospital protocol (Table 2). A clear-film template (Fig. 2) was designed and used to quantify wound ooze as a percentage of the dressing and several shapes of a size of 2 × 2 cm (Fig. 3). Wound ooze of more than 2 × 2 cm at 72 h is considered abnormal and has been shown to be a predictor of post-arthroplasty infection [2, 3].

**Table 1:** Proforma used for data collection

Patient demographics	
ASA	
BMI	
Co-morbidities	
Side of surgery	
Diagnosis	
Antibiotics used	
Wound ooze	
Day 1	
Day 2	
Day 3	
Day 4	
Day 5	
Day 6+	
Number of dressing changes	
Length of stay	
Reason for delayed discharge (≥ 5 days)	
Complications	

*ASA* American Society of Anesthesiologists, *BMI* body mass index

**Table 2:** Hospital’s protocol for wound ooze

**Day 1:** change dressing if soakage > 50%	
**Day 2:** change dressing if soakage > 50%, apply pressure dressing, hold anticoagulation	
**Day 3 (72 h):** change dressing if soakage > 2 x 2 cm, continue to hold anticoagulants, blood investigations (FBC, CRP and clotting)	
**Days 4–6:** discuss with consultant to apply negative pressure dressing PICO® (not used in our study)	
**Day 7**: discuss with consultant regarding early Debridement, Antibiotics and Implant Retention (DAIR) procedure (not encountered in our study)

Patients were discharged only when the wounds were dry and they had passed physiotherapy assessment. The primary outcome measure was the number of dressing changes secondary to wound ooze, whereas the secondary outcome was the LOS and any postoperative complications.

### Statistical analysis

The differences between groups were analyzed with the non-parametric Mann-Whitney *U* test for independent samples using International Business Machines (IBM) Statistical Package for the Social Sciences (SPSS) Statistics 24.

## Results

A total of 43 patients was identified. Tissue adhesive with staples (group 1) was used in 21 patients and staples only (group 2) in 22 patients. The average age for group 1 was 72.2 years (range 54–89) and for group 2 was 70.5 years (range = 50–95), (Table 3).

**Table 3:** Patients’ characteristics

Group/parameter	Group 1 (tissue adhesive) ***n*** = 21	Group 2 (non-tissue adhesive) ***n*** = 22
**Age (years)**		
Mean	72.2	70.5
Range	54–89	50–95
**Gender**		
Male	11	8
Female	10	14
**BMI**		
Median	31.3	30.8
**ASA**		
I	0	2
II	12	12
III	9	8
**Side**		
Right	14	13
Left	7	9
**Surgery**		
Primary	17	19
Revision	4	3
**Diagnosis**		
OA	17	18
Inflammatory, e.g., RA	0	1
Other, e.g., revision	4	3
**Co-morbidities**		
Anticoagulation	4	2
Diabetes	3	1

*ASA* American Society of Anesthesiologists, *BMI* body mass index, *OA* osteoarthritis, *RA* rheumatoid arthritis

**Fig. 4:**
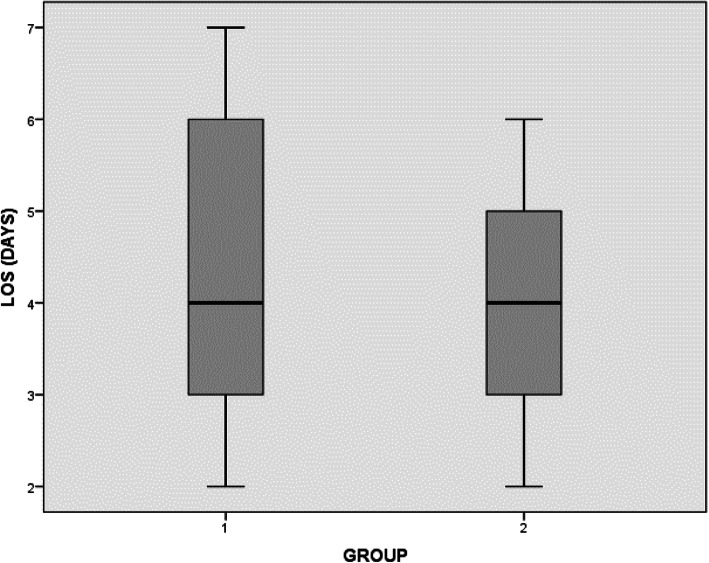
Length of stay in days for both groups (mean, range and confidence interval)

**Fig. 5:**
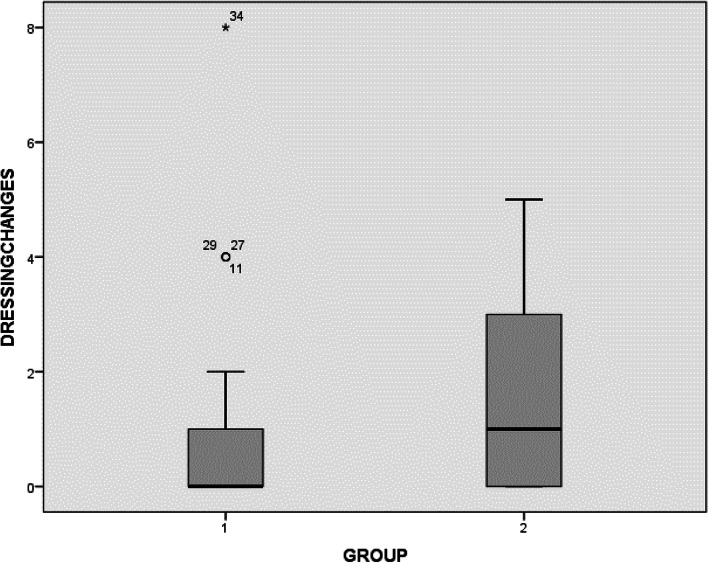
Number of dressing changes for both groups (mean, range and confidence interval)

The tissue adhesive group showed a statistically significant reduction in wound ooze on day 1; however, the difference was not significant on the following days (Table 4), and both groups had equal hospital LOS of an average of 4 days (range of 3–7 days for group 1 and 2–6 days for group 2) (Fig. 4). Dressing changes were less in the tissue adhesive group but did not reach statistical significance (Fig. 5).

**Table 4:** Results of both groups

Group/parameter	Group 1 (tissue adhesive)	Group 2 (non- tissue adhesive)	Significance (***P*** value)
**Mean operative time (min)**	93	90	**Chi-square test. No significant difference between the groups**
**Wound ooze (%)**			
Median			
Day 1	5%	50%	0.019
Day 2	0%	10%	
**Length of stay (days)**			
Median	4	4	0.960
Range	3–7	2–6	
**Dressing changes (total)**			
Median	0	1	0.112
Range	0–8	0–5	
**Prolonged ooze** **(> 72 h)**	4 (19%)	8 (36%)	**Chi-square test. No significant difference between the groups**
**Delayed discharge** **(≥ 5 days)**	8 (38%)	6 (27%)	Not appropriate to analyze
For ooze	3 (14%)	3 (13.5%)	
Physiotherapy/occupational therapy issues	5 (24%)	3 (13.5%)

The longest LOS was 7 days and was observed in four patients, all belonging to the wound adhesive group, but only one was due to wound ooze. The largest number of dressing changes was eight, which was in the wound adhesive group. This patient was a female, with ASA 2, body mass index (BMI) 31, who was taking oral anticoagulant for atrial fibrillation. The wound dried up completely on the day before the patient was discharged and did not develop any wound complications during her hospital stay.

One patient from the staples-only group was readmitted 7 days post-operatively for suspicion of wound infection, which was excluded clinically and by laboratory investigations. This patient was discharged without needing any intervention. We did not encounter any cases of wound dehiscence or any reaction to clips or tissue adhesive in our study.

## Discussion

The anterior midline skin incision used in TKA is subjected to tensile stresses during rehabilitation; therefore, tissue adhesive (LiquiBand®, Advanced Medical Solutions Ltd., Winsford, UK) did not prevent ooze later on during the hospital stay and did not provide any additional benefit to the patients.

**Fig. 6:**
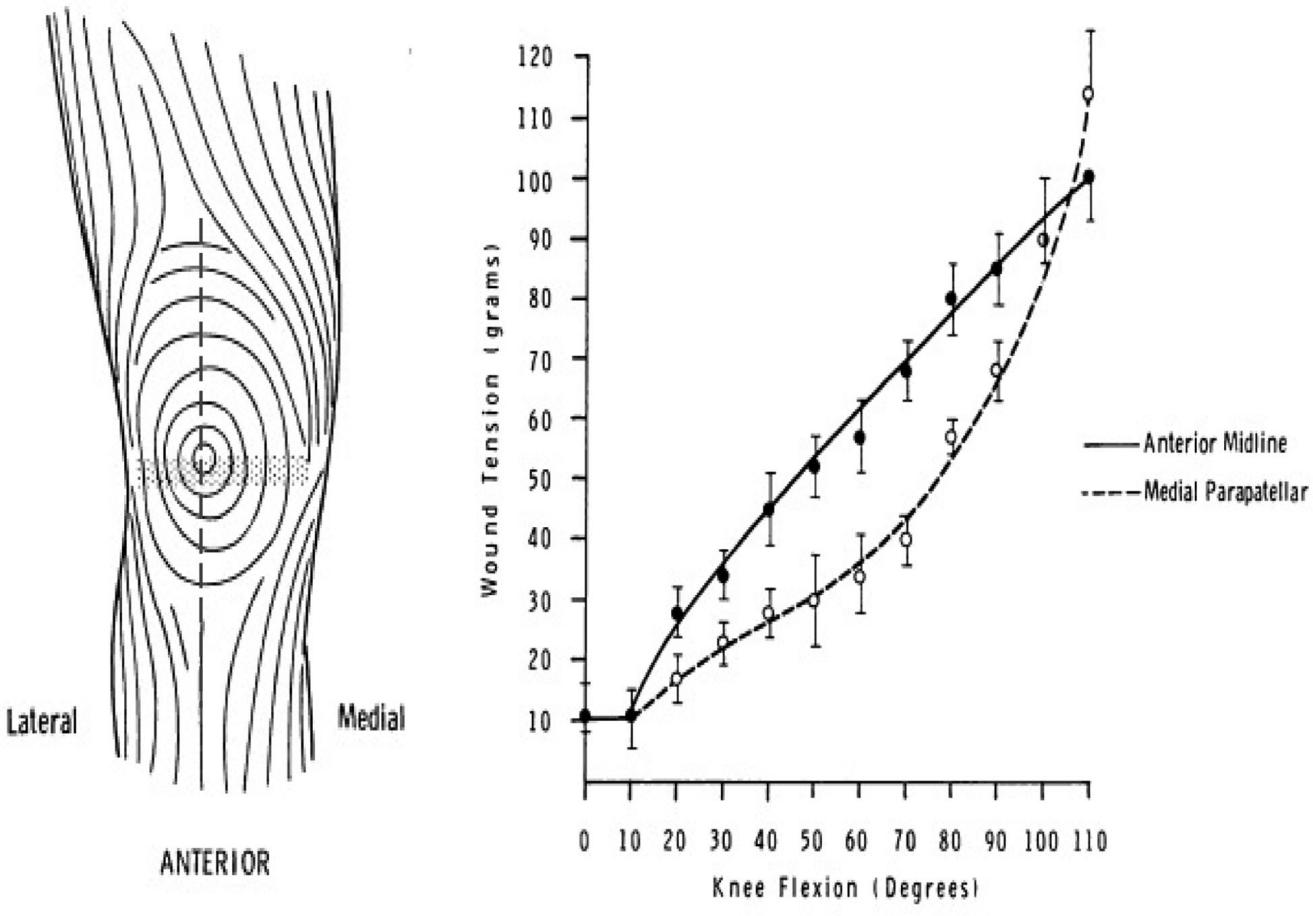
Schematic diagrams showing orientation and forces across anterior midline knee skin incision [17, 18] Left: Midline skin incision is cutting Langer's lines at 90°. Right: High forces on the anterior midline incision with knee flexion compared to the medial parapatellar incision

A classic work by Johnson et al. [17, 18] showed that the anterior midline skin incision is oriented perpendicular to the skin cleavage lines along most of its length and during knee flexion the incision is subjected to significant tension (Fig. 6), which can explain the ooze on the subsequent days.

Many studies analyzing tissue adhesives for surgical wound closure have reported higher wound complications, primarily dehiscence, associated with the wound adhesive. Khan et al. [14] suggested that the initial bond between the tissue adhesive and skin edges was not able to withstand the early rehabilitation characteristic of high-tensile-strength areas such as in knee replacement. Shapiro et al. [11] caution the use of adhesives for high-tension body areas, as do Doyle et al. [19] and Switzer et al. [20]. In fact, most surgery-based studies argued against the use of tissue adhesives for high-stress joints [16].

More recent studies in the literature compared the use of tissue adhesive alone against sutures or staples in THA and TKA. The authors concluded that forces across the knee disrupt the seal by the tissue adhesive and, thus, precipitates ooze, but no wound breakdown was experienced due to meticulous subdermal closure [14, 16]. They concluded that there were few differences in outcomes between all groups. Khan et al. suggested the use of staples alone for all their THA and TKA patients, whereas Eggers et al. found that wound adhesive along with 50% higher-frequency interrupted subcutaneous sutures was the most cost-efficient method for rapid-discharge TKA cases.

Our median LOS of 4 days is less than in the series by Khan et al. [14] with 7 days as median. Eggers el al [16]. showed that the tissue adhesive group had a median stay of 30 h compared to 40 h in the staples group, but this was performed as part of a day-case joint replacement pathway.

There is only one study in the literature that compared the use of tissue adhesive (2-octyl cyanoacrylate (OCA), Dermabond®, Ethicon, Weymouth, UK) as supplement to clips in TKA. Elgazzar et al. [21] concluded that the application of skin tissue adhesive as an adjunct to deep suture and skin staples in TKA resulted in a statistically significant decrease in the immediate post-operative wound ooze on days 2 and 3 as opposed to on day 1 in our study, but at an increased cost which they believed was compensated for by reduced hospital stay. When comparing our study to the work of Elgazzar et al., they had a total of 46 patients split between both groups (21 and 25 patients) and performed randomization and matching between both groups. They used a novel method to quantify and measure the amount of ooze in the post-operative period using a stacked-dressing method. They recorded wound ooze by placing a standardized graph paper over each layer of gauze and recorded the number of boxes of graph paper overlapping any drainage stain on the gauze. The number of drainage boxes for each layer of gauze was added up to quantify the volume of drainage.

We assessed wound ooze until it stopped, whereas Elgazzar et al. did not follow the wound discharge beyond 3 days.

We did not experience any wound complications including infections, wound dehiscence and need for re-operations for prolonged ooze in our study, which shows that the combination of surgery without tourniquet, meticulous operative and hemostatic techniques, tranexamic acid administration and sequential water-tight closure with skin staples to the skin is a reliable method of performing TKA, regardless of the application of wound adhesive.

There was significant reduction of wound ooze on day 1 in the tissue adhesive group compared to the control; however, this did not result in a difference in post-operative wound infection rates. There is no conclusive evidence linking abnormal wound ooze on day 1 with post-operative infection; however, wound ooze beyond 72 h is considered abnormal and a predictor of wound infection after joint arthroplasty [2].

Our study strengths are the use of a single-blinded observer and with an objective method in measuring wound ooze. However, it is not without limitations including non-randomization, a relatively small sample size, use of a single surgeon, potential confounders not being matched between both groups, and a non-validated technique for measuring wound ooze.

## Conclusions

Our series of TKAs suggested that the use of tissue adhesive may reduce wound ooze on day 1, but the reduction observed on days 2 and 3 did not reach statistical significance. Based upon our results, we are not able to recommend the use of a tissue adhesive to reduce wound ooze in TKA, as the significant tensile forces in the immediate post-operative rehabilitation makes its use inappropriate. Further, the cost of tissue adhesive is not offset by reduced dressing changes and length of hospital stay. Future randomized controlled studies need to be conducted with larger patient groups, with the exclusion of potential confounders and with the use of a validated technique of measuring wound ooze.

## Data Availability

All data and materials are available and stored securely onsite for research purposes for up to 5 years. This paper has not been published previously and is not under consideration for publication elsewhere. It will not be published elsewhere in any form or in any other language, without the written consent of the copyright-holder.

## Abbreviations

THA: Total hip arthroplasty
TKA: Total knee arthroplasty
BCA: Butyl cyanoacrylate
TEDs: Thrombo-embolic deterrant stockings
ROM: Range of motion
IBM: International Business Machines
SPSS: Statistical Package for the Social Sciences
LOS: Length of stay
ASA: American Society of Anesthesiologists
BMI: Body mass index
OCA: 2-octyl cyanoacrylate
